# Packaging of actin into Ebola virus VLPs

**DOI:** 10.1186/1743-422X-2-92

**Published:** 2005-12-20

**Authors:** Ziying Han, Ronald N Harty

**Affiliations:** 1 Department of Pathobiology, School of Veterinary Medicine, University of Pennsylvania, 3800 Spruce St., Philadelphia, PA 19104 USA

## Abstract

The actin cytoskeleton has been implicated in playing an important role assembly and budding of several RNA virus families including retroviruses and paramyxoviruses. In this report, we sought to determine whether actin is incorporated into Ebola VLPs, and thus may play a role in assembly and/or budding of Ebola virus. Our results indicated that actin and Ebola virus VP40 strongly co-localized in transfected cells as determined by confocal microscopy. In addition, actin was packaged into budding VP40 VLPs as determined by a functional budding assay and protease protection assay. Co-expression of a membrane-anchored form of Ebola virus GP enhanced the release of both VP40 and actin in VLPs. Lastly, disruption of the actin cytoskeleton with latrunculin-A suggests that actin may play a functional role in budding of VP40/GP VLPs. These data suggest that VP40 may interact with cellular actin, and that actin may play a role in assembly and/or budding of Ebola VLPs.

## Introduction

Ebola virus VP40 is known to bud from cells as a virus-like particle (VLP) independent of additional virus proteins [1-4]. The most efficient release of VP40 VLPs requires both host proteins (*e.g*. tsg101 and vps4), as well as additional virus proteins (*e.g*. glycoprotein [GP] and nucleoprotein [NP]) [5-7]. Cytoskeletal proteins have also been implicated in assembly and budding of various RNA-containing viruses [8-22]. Thus, we sought to determine whether cellular actin may be important for Ebola virus VP40 VLP budding.

## Results

**Figure 1:**
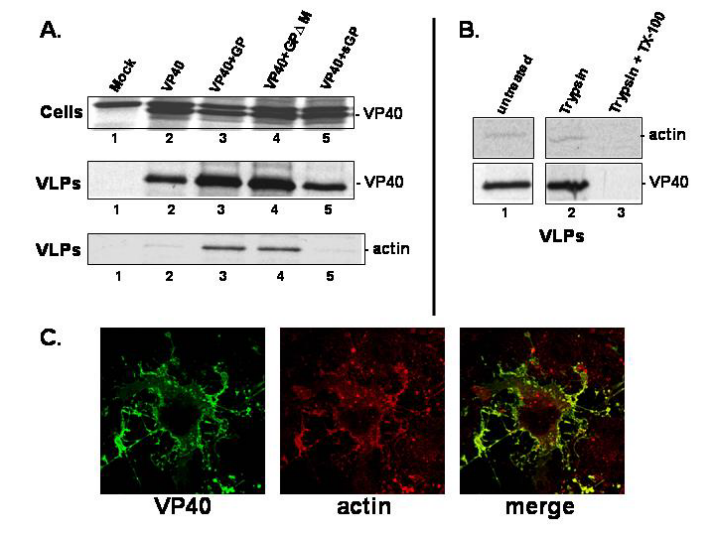
Packaging of actin into VLPs. **A) **Human 293T cells were mock-transfected (lane 1), or transfected with VP40 alone (lane 2), VP40 + GP (lane 3), VP40 + GPΔM (lane 4), or VP40 + sGP (lane 5). Radiolabeled VP40 was detected in cell extracts (cells) and in VLPs. Actin was detected in VLPs by immunoprecipitation using an anti-actin polyclonal Ab. **B) **VP40 VLP samples were untreated (lane 1), treated with trypsin alone (lane 2), or treated with trypsin + TX-100 (lane 3). VP40 and actin were detected by immunoprecipitation. **C) **Indirect immunofluorescence of VP40 (green) and actin (red) with the merged image shown in yellow.

First, we sought to detect actin in budding VP40 VLPs. Human 293T cells were mock-transfected, or transfected with VP40 alone, VP40 + GP, VP40 + a mucin domain deletion mutant (GPΔM), or VP40 + secreted GP (sGP) (Fig. 1A). VP40 synthesis in all cell extracts is shown as an expression control (Fig. 1A, cells). As expected, VP40 alone was readily detected in budding VLPs; however, actin was weakly detectable in VLPs containing VP40 alone (Fig. 1A, VLPs, lane 2). Co-expression of either full-length wild type GP (lane 3), or GPΔM (lane 4) resulted in enhanced release of VP40. Similarly, release of cellular actin was also enhanced in VP40 VLPs containing full-length GP (lane 3), or GPΔM (lanes 4). In contrast, co-expression of sGP (lane 5) did not enhance release of either VP40 or actin (compare lanes 2 and 5). Both VP40 and actin were enhanced 5–6 fold (determined by phosphoimager analysis) in VLPs when GP or GPΔM were co-expressed along with VP40 compared to that when VP40 was expressed alone (data not shown). These results suggest that actin can be packaged in budding VP40 VLPs, and that co-expression of a membrane-anchored form of GP equally enhances release of both VP40 and actin. In addition, GP-mediated enhancement of VP40 VLP budding and actin packaging into VLPs is independent of the mucin-like domain of GP.

To confirm that actin was indeed incorporated into VP40/GP VLPs and does not represent a cellular contaminant, protease protection (Fig. 1B) and flotation gradient analyses (data not shown) were performed. Radiolabeled VP40 VLPs were divided into equal aliquots and treated as indicated in Fig. 1B. Following treatment, β-actin and VP40 were detected by immunoprecipitation and analyzed by SDS-PAGE (Fig. 1B). As reported previously [2,3,6], VP40 was only degraded completely by trypsin in the presence of TX-100 (Fig. 1B lane 3). Similarly, actin was also only degraded completely by trypsin in the presence of TX-100 (lane 3). Treatment with trypsin alone was not sufficient to degrade either VP40 or actin (lane 2). These findings indicate that cellular actin is indeed packaged within Ebola virus VLPs. It should be noted that flotation gradients of purified VLPs were also utilized to demonstrate that actin, VP40, and GP co-purified together in the upper fractions (fractions 2 and 3) of the VLP gradient (data not shown). These findings are consistent with those presented above that actin is incorporated into budding VLPs.

We next sought to use immunofluorescence and confocal microscopy to determine whether VP40 colocalized with cellular actin in COS-1 cells (Fig. 1C). VP40 (green) is known to localize to the cell periphery and can be visualized in membrane fragments or blebs (VLPs) being released from the cell (Fig. 1C). Cellular actin (red) was detected by the use of a polyclonal anti-actin antibody (Santa Cruz Biotechnology, Inc.). Upon merging of the two images, VP40 and actin were found to colocalize (yellow) in many of the membrane fragments that likely represent the formation of VLPs (Fig. 1C). These results correlate with those described above to suggest that VP40 may interact with actin, and that actin may be incorporated into budding VLPs in a specific manner.

**Figure 2:**
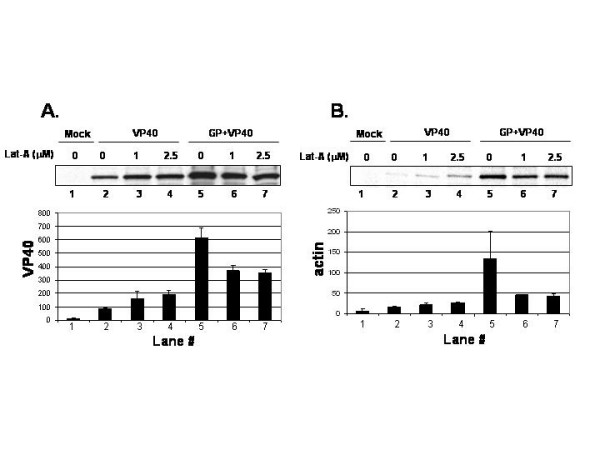
Affect of Latrunculin-A on VLP budding. VLPs were isolated from mock-transfected cells or cells transfected with VP40 alone or VP40 + GP in theabsence, or presence of the indicated concentration of lat-A. VP40 (panel A) or actin (panel B) was detected by immunoprecipitation and quantitated by phosphoimager analysis of at least two independent experiments.

Latrunculin-A, which disrupts actin filaments by binding actin monomers to prevent them from polymerizing, was used to disrupt the actin cytoskeleton. Concentrations of latrunculin-A utilized in these experiments were shown to disrupt actin filaments by immunofluorescence staining (data not shown). Human 293-T cells were transfected with VP40 alone, or with VP40 + full-length GP (Fig. 2). At 24 hours post-transfection, cells were pretreated with or without the indicated concentrations of latrunculin-A for 20 min. and were then radiolabeled with [^35^S]Met-Cys in the presence or absence of latrunculin-A for 5 hours. VLPs and cell extracts were prepared as described above. VP40 (panel A) and actin (panel B) in VLPs were detected by immunoprecipitation and analyzed by phosphor-imager analyses. Interestingly, VP40 VLP release was slightly stimulated in the presence of 1.0 and 2.5 μM latrunculin-A (Fig. 2A, lanes 3 and 4), compared to that in the absence of drug (lane 2). A similar result was observed in the presence of identical concentrations of cytochalasin D (data not shown). In contrast, release of VP40/GP VLPs was slightly reduced in the presence of Lat-A (Fig. 2A, lanes 6 and 7), compared to that in the absence of drug (lane 5). The effect of Lat-A on packaging of actin into VLPs paralleled that of VP40 (Fig. 2B). For example, in the presence of 1.0 and 2.5 μM lat-A, slightly more actin was packaged into VP40 VLPs (Fig. 2B, lanes 3 and 4) than that in the absence of drug (lane 2). In contrast, reduced amounts of actin were packaged into VP40/GP VLPs in the presence of lat-A (Fig. 2B, lanes 6 and 7) than in the absence of drug (lane 5). These results indicate that lat-A partially inhibits both VP40 and actin release in VLPs only when VP40 and GP are co-expressed in cells. However, lat-A treatment slightly enhanced release of VP40 budding alone. Treatment with actin depolymerizing drugs has been reported to both increase and decrease budding of other RNA viruses [9,10,18,23,24].

## Discussion

The mechanism by which GP enhances budding of VP40 VLPs remains unclear [6]. Preliminary data from our lab suggests that GP does not enhance budding of VP40 via a direct protein-protein interaction (data not shown). An alternative possibility is that GP modifies the cell in a global manner that positively influences VP40 release. Indeed, GP is known to be cytotoxic and induces cell rounding and detachment [25-27]. Thus, GP expression likely induces significant changes to the cellular cytoskeleton during infection. Lat-A may be inhibiting the mechanism by which GP enhances budding of VP40 (Fig. 2). It remains to be determined whether actin directly interacts with VP40, or whether actin may directly interact with GP.

The actin cytoskeleton has been implicated in assembly and budding of Newcastle disease virus, HIV-1, Black Creek Canal Virus, fowlpox virus, West Nile virus, equine infectious anemia virus, and respiratory syncytial virus RSV [9,10,14,18,20,23,24]. Cellular actin has been detected in virion or virus-like particles of murine mammary tumor virus (MuMTV), Moloney murine leukemia virus (MoMuLV), HIV-1, and Sendai virus [11,13,15,16,28]. Ebola virus VP40 has recently been shown to associate with microtubules and enhance tubulin polymerization [19]. Yonezawa et al. found that agents that inhibited microfilaments also inhibited entry and fusion of Ebola virus GP pseudotypes [29]. These authors suggest that microtubules and microfilaments may play a role in trafficking Ebola virions from the cell surface to acidified vesicles for fusion.

## Conclusion

Our data indicate that actin is indeed packaged into Ebola virus VLPs. Co-expression of a membrane-anchored form of GP enhances release of actin and VP40 by equivalent levels in VLPs. The mucin-like domain of GP was not necessary for enhancement of VP40 or actin release in VLPs. VP40 was found to co-localize with actin suggesting that VP40 may interact with actin and perhaps may utilize the actin network for assembly and budding VLPs from the plasma-membrane. Lat-A treatment resulted in a slight increase in budding of VP40 VLPs; however, the same concentrations of lat-A resulted in a slight decrease in budding of VP40/GP VLPs. Experiments are now underway to understand further the mechanism of action of lat-A and other actin depolymerizing drugs on Ebola VLP budding. In addition, we will attempt to determine whether actin binding proteins may be involved in VLP budding. Lastly, experiments are underway to determine whether actin plays a role in assembly and budding of live Ebola virus.

## Competing interests

The author(s) declare that they have no competing interests.

## Authors' contributions

ZH performed all of the experiments. ZH and RH contributed to the conception, design, analysis, and interpretation of the data. ZH and RH contributed to the writing of the manuscript.
